# Supplementary material for: MetaRibo-Seq measures translation in microbiomes
Source: Nat Commun. 2020 Jun 29;11:3268. doi: 10.1038/s41467-020-17081-z (PMC7324362; doi:10.1038/s41467-020-17081-z)
Supplement: Supplementary file 10 — Supplementary Data 7 [file 41467_2020_17081_MOESM10_ESM.zip › File2/Confidence_High_Taxonomy/321417_out.krona.html]

Javascript must be enabled to view this page.

members
magnitude
magnitudeUnassigned
count
unassigned
taxon
rank

321417\_out

5

superkingdom
5
2

5
phylum
1239

186801
1
class

186802
1
order

186806
family
1

1730
genus
1

species
1

SRS015431\_contig\_number\_67324
1262878

class
4
909932

909929
order
4

family
4
1843491

970
4
genus

species
1

SRS058808\_contig\_number\_22161
712532

1321785

SRS104539\_contig\_number\_27446
1
species

species
2

SRS014402\_contig\_number\_24323SRS101445\_contig\_number\_49775
135082
